# Distinct chromosome abnormality patterns for differential diagnosis of hepatocellular carcinoma and cholangiocarcinoma

**DOI:** 10.1371/journal.pone.0322408

**Published:** 2025-05-12

**Authors:** Wantakan Ngamsangiam, Sutheemon Techa-ay, Prakasit Sa-ngiamwibool, Sasithorn Watcharadetwittaya, Raksawan Deenonpoe, Anchalee Techasen, Natruja Sridakhun, Sureerat Padthaisong, Malinee Thanee

**Affiliations:** 1 Department of Pathology, Faculty of Medicine, Khon Kaen University, Khon Kaen, Thailand; 2 Cholangiocarcinoma Research Institute, Khon Kaen University, Khon Kaen, Thailand; 3 Faculty of Associated Medical Science, Khon Kaen University, Khon Kaen, Thailand; 4 Faculty of Allied Health Sciences, Burapha University, Chonburi, Thailand; University of Surrey, UNITED KINGDOM OF GREAT BRITAIN AND NORTHERN IRELAND

## Abstract

Hepatocellular carcinoma (HCC) and cholangiocarcinoma (CCA) are primary liver cancers with overlapping histopathological features, making accurate diagnosis challenging. This study aimed to identify chromosomal abnormalities that could aid in differentiating these cancers using chromosome microarray analysis (CMA). We analyzed ten frozen tumor tissues each of HCC and CCA, identifying distinct patterns of chromosomal gains and losses. HCC exhibited gains in regions 1p36.32, 1q23.3-q24.1, 3q21.3, 4p16.1, 5q31.1, and 11p15.5, and losses in 2p15, 3p11.1-q11.1, 4q12, 5p12-q11.1, 7q11.23, 14q23.2, 17p11.2, 17p13.3, 22q12.1, 22q12.2-q12.3, and 22q13.2. In contrast, CCA showed gains in 5p13.2, 5p14.1, 8p12-p11.23, 8p22, and 19p13.2, and losses in 1q31.1, 1q42.13, 3p25.3, 6p12.1, 6p25.3, and 17q21.33. Heatmap analysis revealed 17 distinct chromosomal regions between the two groups including 2q14.2, 4p16.3, 5q32, 7p14.3, 7p22.1, 7q11.21, 7q11.23, 7q21.3, 7q22.1, 10q21.3, 18q23, 19p13.2, 19q13.2, 21q21.3, 21q22.13, 22q11.21, and 22q12.2. Among these 1p36.32, 19p13.2, and 19q13.2 emerged as potential biomarkers for differential diagnosis. These findings may aid in confirming cases with overlapping histopathological features contribute to the development of diagnostic tools and improved targeted therapies for HCC and CCA.

## 1. Introduction

Primary liver cancer is a significant global health burden, with hepatocellular carcinoma (HCC) and cholangiocarcinoma (CCA) as the two most common subtypes. It is the sixth most common cancer worldwide and the fourth leading cause of cancer related death in 2020 with approximately 906,000 new cases and 830,000 deaths by GLOBOCAN [1]. HCC is the most common primary liver cancer, comprises 75%-85% of cases and originates from the hepatocytes [2]. While, CCA is a secondary common primary liver cancer (10%-15%) emerging in the biliary tree, and frequently found in northeastern Thailand [3]. Hepatitis B virus (HBV) followed by hepatitis C virus (HCV) are the major etiologic risk factor for HCC across all regions of Thailand [4]. In contrast, the primary rick factor for CCA in Northeast Thailand particularly in Khon Kaen is liver fluke infection (*Opisthorchis Viverrini*), which accounts for approximately 89% of all liver cancers in this region [5]. In addition, CCA can be classified into three subtypes based on their anatomical site of origin as intrahepatic CCA (iCCA), perihilar CCA (pCCA) and distal CCA (dCCA) [3]. While both originate in the liver, they exhibit distinct histopathological features and treatment requirements. However, the overlapping etiologies and histopathological features of HCC and CCA, as seen in some cases (e.g., hepatitis B/C in CCA cases) [6–8], highlight the need for novel diagnostic tools to identify biomarkers, could complement existing methods, improving diagnostic accuracy in challenging cases and potentially enabling targeted therapies. In clinical practice, non-surgical methods are commonly employed for initial diagnostic testing. These include liver function tests, tumor markers, and imaging techniques such as computed tomography (CT) and magnetic resonance imaging (MRI). Alpha-fetoprotein (AFP) is a widely used tumor marker for HCC [9]. Patients with abnormal surveillance findings such as liver nodules detected by abdominal ultrasonography or elevated AFP levels are typically further evaluated with CT or MRI [10]. In contrast, the tumor markers such as carbohydrate antigen (CA) 19–9, and carcinoembryonic antigen (CEA) are often used for CCA diagnosis [11]. When these markers are inconclusive, imaging techniques such as ultrasonography, CT, contrast-enhanced ultrasonography (CEUS), MRI and Magnetic resonance cholangiopancreatography (MRCP) are generally recommended [12]. Accurate diagnosis is crucial for appropriate management, but traditional methods like hematoxylin and eosin (H&E) staining and immunohistochemical (IHC) analysis can be challenging due to overlapping features and potential limitations in marker sensitivity and specificity [13]. Arginase-1 and HepPar-1 are established immunostaining markers for identifying hepatocellular cell origin, as demonstrated in previous studies. CK7 and CK19 are expressed in cholangiocytes. These markers aid in differentiating hepatocellular carcinoma (HCC) from metastatic carcinoma and cholangiocarcinoma (CCA). In diagnostically challenging cases of HCC versus CCA, pathologists may use a combination of HepPar-1, Arginase-1, CK7, and CK19 [14]. They reported the sensitivity of Arginase-1 and HepPar-1 to be 84% and 70%, respectively, while their specificity was 96% and 84%, respectively. These findings suggest that HCC markers may show positive results in CCA, and conversely, CCA markers may be positive in HCC cases, highlighting the potential limitations of these markers in differentiating between the two cancers [14,15].

Recent advancements in high-throughput molecular analysis have enabled the identification of molecular alterations, including genomic mutations and chromosomal abnormalities (also referred to as chromosomal aberrations which encompass both numerical and structural changes in chromosomes) such as amplifications (commonly known as gains occur when regions of a chromosome are duplicated resulting in the extra genetic material and leading to increased protein production), deletions (also referred to losses where a portion of a chromosome is missing or deleted resulting in the loss of one or more genes), translocations, and other structural variations, which are frequently associated with cancer development and progression [16–18]. These insights can inform the development of molecularly targeted therapies [16]. Furthermore, the application of this approach may be beneficial for differentiating between HCC and CCA. Previous studies comparing genetic alterations between HCC and iCCA using comparative genomic hybridization (CGH) identified distinct chromosomal patterns. HCC was characterized by gains in chromosome region 1q and losses in regions 1p, 4q, 10q, and 13q. In contrast, iCCA exhibited gains in regions 5p, 7p, 13q, and 20q. However, both cancers shared similar patterns of losses in regions 16q, 17p, and 18q, as well as gains in region 8q [19]. While comparative genomic hybridization (CGH) was previously used to study genetic alterations, it has largely been replaced by higher-resolution technologies capable of detecting chromosomal abnormalities throughout the whole genome. Chromosome microarray analysis (CMA) is one such high-throughput technique that can identify various chromosomal abnormalities, including gains and losses. It has a high sensitivity of genome-wide resolution enabling the detection of chromosomal abnormalities, down to a 50–100 kb level across the genome [20]. These abnormalities are presented as log2 ratios and copy number variations (CNVs refer to duplicated or deleted DNA segments at least 1,000 base pairs in size that differ from the reference genome) [21–23]. Compared to other high-throughput techniques such as next-generation sequencing (NGS) which is designed for detecting single-nucleotide polymorphisms and small insertions/deletions [24]. CMA offers a more cost-effective and time-efficient approach for detecting large CNVs, aneuploidies and loss of heterozygosity (LOH) with high accuracy, unlike NGS which requires extensive library preparation, sequencing and bioinformatics analysis [23,25]. Additionally, CMA generates less complex data that facilitates interpretation. In addition, CNVs have previously been correlated with clinical features and may be used for predicting prognosis. In HCC patients, studies have shown that loss of region 8p is associated with a poor prognosis [26]. Additionally, the previous study has showed that fluorescence in situ hybridization (FISH) can be used to detect several chromosomal abnormalities, including polysomy signals of chromosomes 7, 17, and 7/17, which have been associated with a poor prognosis in CCA patients [27]. Interestingly, a comparative analysis of copy number alterations and transcriptomics in iCCA and HCC with similar molecular subtypes identified 51 genes that were commonly dysregulated in both cancer types. However, 188 genes were uniquely dysregulated in iCCA, while 38 were uniquely dysregulated in HCC, suggesting distinct molecular subtypes within each cancer type [28].

This study aims to investigate the chromosomal patterns in HCC and CCA to identify potential biomarkers that can aid in their differential diagnosis. By understanding the distinct genomic landscapes of these cancers, we may be able to improve diagnostic accuracy and inform targeted therapeutic approaches.

## 2. Materials and methods

### 2.1 Patient and sample collection

Frozen liver tumor tissues from 10 patients with HCC and 10 patients with cholangiocarcinoma CCA and their clinicopathological data were obtained from Srinagarind Hospital and stored at the Cholangiocarcinoma Research Institute, Khon Kaen University, Thailand. These patients underwent surgery prior to treatment and were diagnosed with HCC or CCA by a pathologist. The frozen tissue samples and clinicopathological data (sex, age, diagnosis, tumor size) of CCA patients from January 1, 2017, to May 30, 2023, were assessed and collected on October 1, 2023, and finished on December 31, 2023. Tumor purity was assessed using H&E staining by an experienced pathologist to determine the percentage of tumor cells relative to the total cellular content. A tumor purity of at least 70% was achieved for each patient according to the proposed by Tapial et al. [29]. To ensure participant anonymity during data collection, the Cholangiocarcinoma Research Institute blinded identifiable information by replacing hospital numbers (HN) and other identity-related data with unique index numbers. As a result, the authors had access only to de-identified information, preventing the identification of individual participants. Patient characteristics are summarized in Table 1. Additionally, the etiologies involved in the development of HCC and CCA for each patient are provided in S1 Table.

**Table 1:** Characteristics of primary liver cancer patients.

	HCC (%) (n = 10)	CCA (%) (n = 10)
Age (years)	56 (range: 45–75)	64 (range: 58–79)
Sex		
Male	8 (80%)	8 (80%)
Female	2 (20%)	2 (20%)
Tumor size (cm)	6.7 (range: 4–11)	10 (range: 7–15)

HCC: Hepatocellular carcinoma, CCA: Cholangiocarcinoma.

#### 2.1.1 Ethics statement.

This study was conducted in accordance with the Declaration of Helsinki and the International Council for Harmonization (ICH) Good Clinical Practice Guidelines.

It was approved by the Center for Ethics in Human Research at Khon Kaen University (HE661431). Given the retrospective nature of the data collection, the anonymization of patient information, and the minimal risk involved, informed consent was not required. This exemption was approved by the Khon Kaen University Ethics Committee for Human Research.

### 2.2 Genomic DNA extraction

Genomic DNA was extracted from tumor tissues (25–35 mg) using the QIAmp DNA mini kit (Qiagen, Hilden, Germany). Tissue samples were lysed in buffer containing proteinase K and incubated at 56 °C for 2–3 hours. After adding buffer and incubating at 70 °C for 10 minutes, absolute ethanol was added, and the supernatant was transferred to a column filter tube and centrifuged. The column was washed with 500 µ L of buffer and centrifuged again. The DNA was eluted with buffer in a microcentrifuge tube and centrifuged for 1 minute at 6000 g. DNA concentration was measured using a NanoDrop spectrophotometer, and all genomic DNA samples were stored at -20 °C for future use.

### 2.3 Chromosome microarray technique

Chromosome microarray analysis was conducted using the CytoScanTM 750K platform (Thermo Fisher Scientific, Waltham, MA, USA). The CMA laboratory workflow comprised nine steps: digestion, ligation, polymerase chain reaction (PCR) setup, PCR purification, quantitation, fragmentation, labeling, hybridization, and wash-stain-scan. Initially, genomic DNA from patient samples was digested with Nsp I. Subsequently, adapters were ligated to the restriction fragments and amplified using PCR. PCR products were purified using magnetic beads, and the yield was measured using Nanodrop. The purified DNA was fragmented into short strands and labeled with biotin. Labeled DNA was hybridized to an AFFYMETRIX™ GeneChip™ microarray for 16–18 hours in a hybridization oven. After hybridization, the array chips were washed, stained with streptavidin, and scanned using a GeneChip Scanner to generate CEL files containing probe intensity signals. Computer analysis was then used to compare the signal intensity changes from the patient samples with the database of genomic DNA from healthy individuals. Log2 ratios and copy number states were calculated to identify chromosomal abnormalities using the Affymetrix Chromosome Analysis Suite Software (ChAS).

### 2.4 Chromosome data analysis and statistical analysis

**Fig 1:**
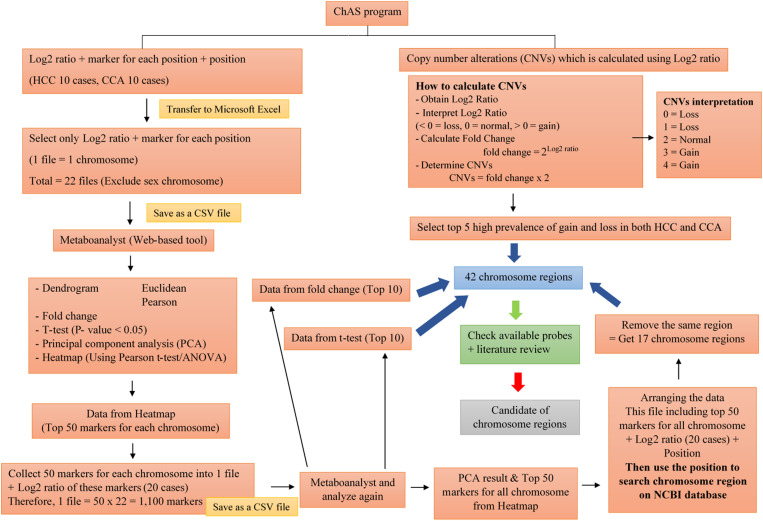
Workflow for chromosome data analysis. ChAS data was transferred to Excel, arranged, and saved as CSV files. These files were imported into bioinformatics tools for analysis. A heatmap was generated to visualize the top 50 probe markers on each chromosome. The results were combined into a single CSV file and analyzed using Principal Component Analysis (PCA) to identify group differences. The heatmap was also used to analyze the top 50 regions across all chromosomes. Candidate chromosome abnormality regions were identified based on the top 10 log2 fold change, top 10 p-value, and top 5 prevalence of loss and gain in HCC and CCA patients.

The Affymetrix Chromosome Analysis Suite Software (ChAS) generated data on chromosomal patterns, including copy number states, log2 ratios, and visualizations of whole genome views and karyoviews. To classify groups of chromosomal patterns based on log2 ratios, principal component analysis (PCA) and heatmap analysis were performed using bioinformatics tools (Metaboanalyst 4.0). PCA was conducted with autoscaling as the data normalization method and the first two principal components were used for visualization. Heatmaps were generated to analyze the top 50 regions across all chromosomes using Pearson’s correlation coefficient as the distance metric and Ward’s linkage method for hierarchical clustering. Chromosomal positions were aligned with the GRCh37/hg19 genomic version. Additionally, these results were integrated with the top 10 fold change values, top 10 p-values (p < 0.05), and the top 5 most prevalent regions of loss and gain in HCC and CCA patients (CNVs data) to identify candidate chromosomal abnormality regions. The independent t-test was used to compare the copy number ratios between HCC and CCA patients. A p-value < 0.05 was defined as statistically significant. The chromosome data analysis workflow is illustrated in Fig 1.

### 2.5 Fluorescence in situ hybridization assay

Fluorescence in situ hybridization (FISH) was performed on formalin-fixed paraffin-embedded (FFPE) tissue sections to validate candidate chromosomal abnormality regions, using samples with known copy number variation (CNV) profiles from chromosome microarray analysis (CMA). Four-micrometer sections were cut from FFPE blocks, baked at 60 °C overnight, deparaffinized in xylene, and dehydrated in 100% ethanol. Slides were pretreated with SSC buffer (Abbott Molecular, USA), rinsed with purified water, and then incubated in 50 mL protease buffer containing 100 mg pepsin. Following dehydration in a graded ethanol series (70–100%), a 1p36/1q25 dual-color probe set (Vysis/Abbott Molecular Inc.) was applied. The 1p36 probe spans from near the SHGC57243 locus, covering the TP73 and MEGF6 genes, to the RH75821 loci. The 1q25 probe spans from the WI-6848 locus, through the ABL2 and ANGPTL1 genes, to near SHGC-1322. Probes were hybridized to slides on a ThermoBrite at 73 °C for 5 minutes, followed by overnight incubation at 37 °C. Slides were then washed with 2X SSC/0.3% Tween 20, counterstained with 4’,6-diamidino-2-phenylindole (DAPI), coverslipped, and stored at -20 °C in the dark. Probe signals were analyzed via fluorescence microscopy, with two signals indicating normal chromosomes, three or more signals indicating gain, and one or no signal indicating loss.

## 3. Results

### 3.1 The pattern of chromosome aberration in HCC patients

**Fig 2:**
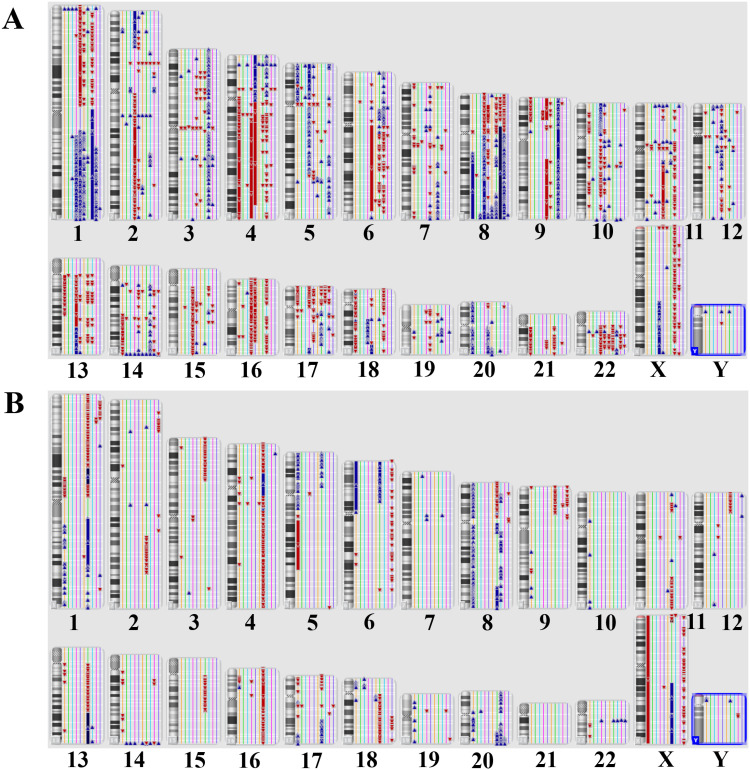
The karyoview analysis using ChAS software. The figure presents an ideogram of all copy number variation in HCC cases (A) and (B) CCA cases. Blue bar represents gain; red bar represents loss.

The most common chromosomal aberration observed in HCC was the loss of chromosome 22q12.1, occurring in 7 of 10 cases (70%) as revealed by karyoview analysis (Fig 2A) and whole genome view (S1 Fig). Recurrent chromosomal abnormalities included gains of chromosome 1p36.32 (60%), 3q21.3 (60%), and 4p16.1 (60%) (Table 2). Additionally, gains of chromosome 11p15.5 (50%), 5q31.1 (40%), and 1q23.3-q24.1 (40%) were frequently observed. Furthermore, losses at 2p15, 3p11.1-q11.1, and 17p11.2 occurred in 6 of 10 cases (60%), while losses at 4q12, 5p12-q11.1, 7q11.23, 14q23.2, 17p13.3, 22q12.2-q12.3, and 22q13.2 were seen in 5 of 10 patients (50%).

**Table 2:** Recurrence of chromosome abnormality in HCC.

	Gain in HCC	%	Loss in HCC	%
22q12.1	1	10	7	70
2p15	0	0	6	60
3p11.1q11.1	0	0	6	60
17p11.2	0	0	6	60
4p14	0	0	5	50
4q12	0	0	5	50
4q13.2	0	0	5	50
5p12q11.1	0	0	5	50
7q11.23	0	0	5	50
11p11.12q11	0	0	5	50
14q12	1	10	5	50
14q23.2	0	0	5	50
17p13.3	0	0	5	50
17q12	0	0	5	50
22q12.2q12.3	0	0	5	50
22q13.2	0	0	5	50
1p36.32	6	60	1	10
1q32.1	5	50	0	0
2q14.2	6	60	0	0
3q21.3	6	60	0	0
4p16.1	6	60	0	0
8q24.3	6	60	0	0
11p15.5	5	50	0	0
1q23.3q24.1	4	40	0	0
5q31.1	4	40	0	0

### 3.2 Chromosomal abnormalities of CCA patients

Chromosomal aberrations in CCA patients were highly diverse and individualized. Karyoview analysis (Fig 2B) and whole genome view (S2 Fig) revealed the most prevalent chromosomal aberrations in CCA patients is gain of 22q11.22 in 5 of 10 cases (50%). Additionally, the recurrence gains of 1q32.2, 8q12.1, 20p11.21, 20q13.33 (3 cases, 30%), 2q14.2 and 8q24.3 (2 cases, 20%) were predominant, whereas loss at 17q12 are most seen in 4 of 10 cases (40%). The losses at 4q13.2 and 9p24.3 were also found in 3 of all 10 patients (30%) (Table 3).

**Table 3:** Recurrence of chromosome abnormality in CCA.

	Gain in CCA	%	Loss in CCA	%
22q11.22	5	50	0	0
1q32.2	3	30	0	0
8q12.1	3	30	0	0
20p11.21	3	30	0	0
20q13.33	3	30	0	0
2q14.2	2	20	0	0
8q24.3	2	20	0	0
17q12	0	0	4	40
4q13.2	0	0	3	30
9p24.3	0	0	3	30
				
1p21.1p13.3	0	0	2	20
1p13.3p13.2	0	0	2	20

### 3.3 The different patterns of chromosome abnormalities in HCC and CCA patients

**Fig 3:**
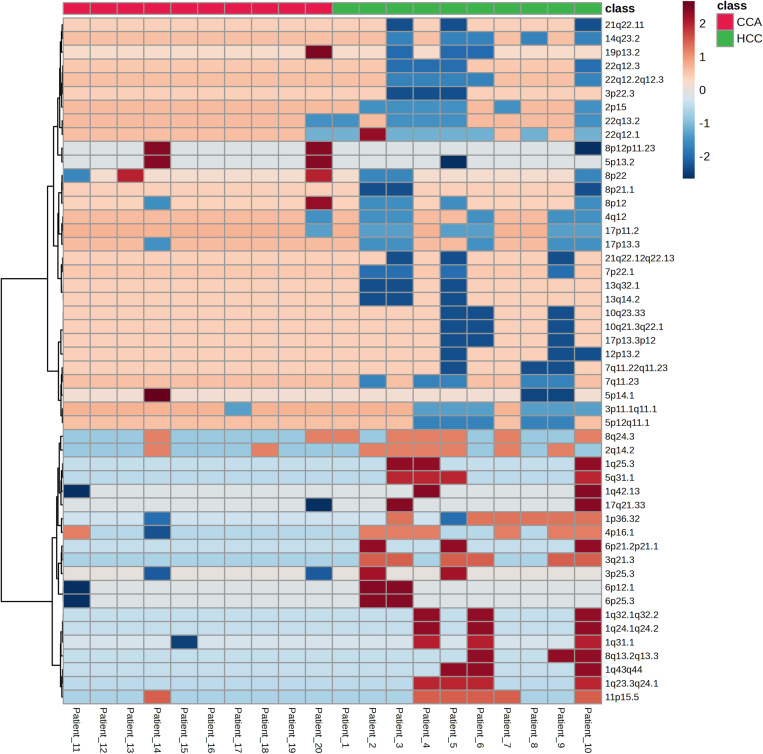
A heatmap clustering of chromosomal abnormality regions. The x-axis represents the number of cases with hepatocellular carcinoma (HCC) and cholangiocarcinoma (CCA). The y-axis lists the top 50 chromosomal abnormality regions. Z-scored copy number variations (CNVs) range from -2 to 2. Red indicates CNV gains, while blue indicates CNV losses.

Despite distinct chromosomal aberration patterns between HCC and CCA, both cancers exhibited gains at 2q14.2 and 8q24.3 (Fig 3). To further characterize these alterations, we analyzed the frequency of CNVs across all chromosomal regions in HCC and CCA cases (S2 Table). Copy number analysis revealed that HCC frequently exhibited amplifications in 1p36.32, 2q14.2, 3q21.3, 4p16.1, and 8q24.3, while CCA was characterized by gains in 1q32.2, 8q12.1, 20p11.21, 20q13.33, and 22q11.22 (Tables 2 and 3). Additionally, HCC frequently exhibited losses at 2p15, 3p11.1-q11.1, 17p11.2, and 22q12.1, whereas CCA commonly showed deletions at 4q13.2, 9p24.3, and 17q12. Notably, heatmap clustering of chromosomal abnormality regions revealed chromosome gains in CCA patients at 5p13.2, 8p12-p11.23, 8p22 and 19p13.2, while losses at 1q31.1, 3p25.3, and 17q21.33 (Fig 3). Interestingly, the 19p13.2 region showed chromosome gain in CCA patient, whereas chromosome loss was observed in HCC patients.

### 3.4 Discriminating chromosomal patterns in hepatocellular carcinoma and cholangiocarcinoma: A principal component analysis and heatmap approach

**Fig 4:**
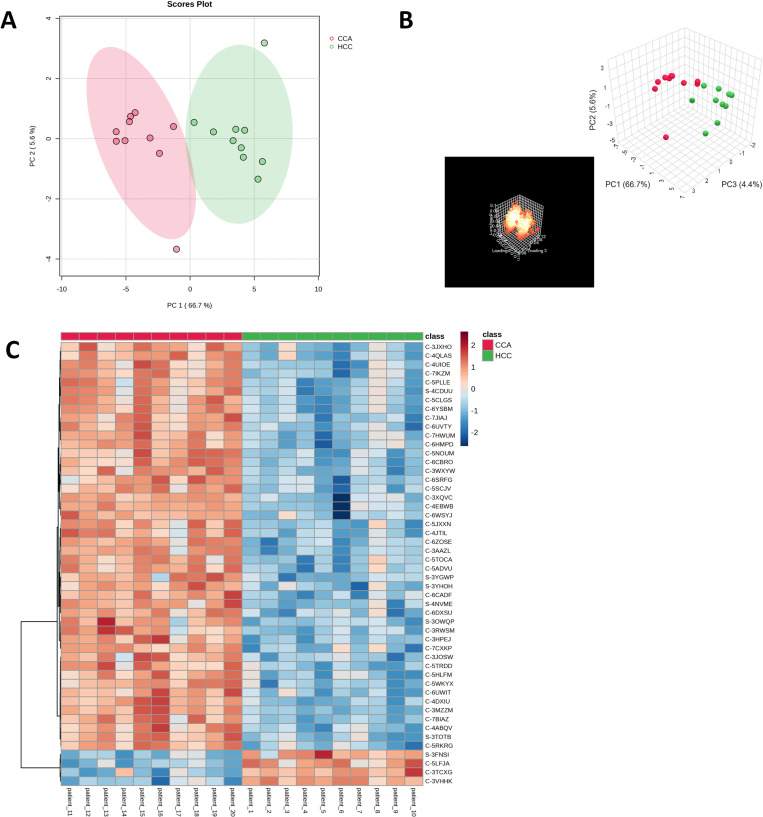
Identification of HCC and CCA chromosome abnormality regions. (A) A principal component (PC) scores plot of HCC and CCA patients. (B) The PC analysis of HCC and CCA. (C) A heatmap clustering chromosome region marker. The x-axis represents cases of HCC and CCA. The y-axis represents different chromosome region marker**s**. Z scored log2 ratio are shown from -2 to 2. Red indicates chromosome gain and blue indicates chromosome loss.

**Fig 5:**
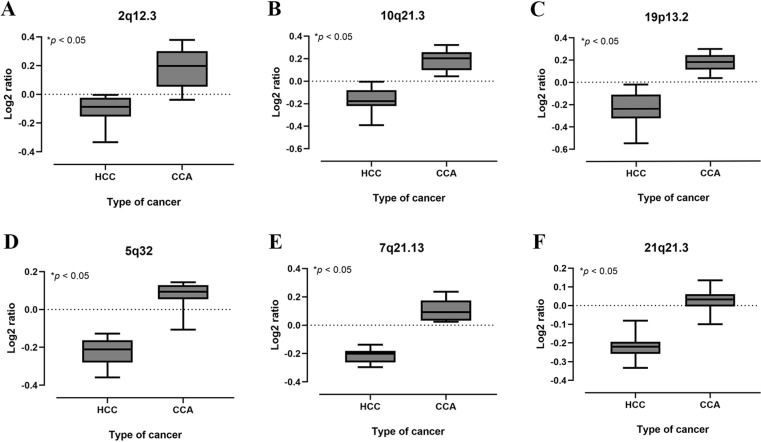
The differential Log2 ratios between HCC and CCA in candidate regions. The graph of log2 ratio of chromosome region 2q12.3 (A), 10q21.3(B), 19p13.2(C), 5q32 (D), 7q21.13 (E) and 21q21.3 (F) in HCC and CCA.

To illustrate the distinct chromosomal patterns between HCC and CCA, principal component analysis (PCA) was employed. The PCA scores plot for all HCC and CCA patients demonstrated clear separation along PC1, with the two principal components accounting for 72.3% of the variance (PC1: 66.7%, PC2: 5.6%) (Fig 4A and 4B). Heatmap analysis of all chromosome probe markers identified 17 regions that significantly differed between HCC and CCA groups. These regions included C-6WSYJ (2q14.2), C-5LFJA (4p16.3), C-6ZOSE (5q32), C-6CADF (7p14.3), C-5HLFM (7p22.1), C-5PLLE(7q11.21), C-3HPEJ (7q11.23), S-3 YHOH (7q21.3), C-3JOSW (7q22.1), C-3JXHO (10q21.3), S-3 FNSI (18q23), C-7HWUM (19p13.2), C-7JIAJ (19q13.2), S-4 NVME (21q21.3), C-6DXSU (21q22.13), C-3XQVC (22q11.21), and C-5ADVU (22q12.2) (Fig 4C). Notably, heatmap analysis identified an elevated signal at 4p16.3 in HCC but a decreased signal in CCA. Furthermore, log2 signals at 7p22.1, 7q11.21, 7q11.23, 7q22.1, 10q21.3, 19p13.2, 19q13.2, 21q21.3, 21q22.13, and 22q11.21 were higher in CCA compared to HCC. Additionally, the fold change (S3 Table) and p-value (S4 Table) of the top 50 probe markers in these regions were analyzed to highlight the differences in signal intensities and CNVs levels between HCC and CCA. The top 3 markers with the highest fold change in CCA were 2q12.3, 10q21.3, and 19p13.2, while HCC exhibited lower signals (Fig 5A–5C, Table 4). Similarly, the top 3 markers with the lowest p-values were 5q32, 7q21.13, and 21q21.3 in CCA, that higher signal than HCC (Fig 5D–5F, Table 5).

**Table 4:** The differential copy number variations ratios between HCC and CCA in the top 10 chromosome region with the highest fold change.

Position	Chromosome region	Human genome version	Fold change (10)	The copy number ratio between HCC and non-tumor samples (Mean±SD)	The copy number ratio between CCA and non-tumor samples (Mean±SD)	*P*-value
109206673	2q12.3	GRCh37/hg19	10355	0.936 ± 0.061	1.089 ± 0.205	0.036*
70376128	10q21.3	GRCh37/hg19	10236	0.950 ± 0.075	1.144 ± 0.180	0.005*
12953399	19p13.2	GRCh37/hg19	9673.3	0.967 ± 0.099	1.212 ± 0.190	0.002*
34915853	7p14.3	GRCh37/hg19	9170.1	0.988 ± 0.066	1.151 ± 0.187	0.018*
49148558	20q13.13	GRCh37/hg19	9055.8	1.078 ± 0.062	1.277 ± 0.250	0.025*
75263013	7q11.23	GRCh37/hg19	8932.2	0.944 ± 0.046	1.113 ± 0.183	0.011*
74646149	11q13.4	GRCh37/hg19	8904.2	0.865 ± 0.103	1.037 ± 0.155	0.009*
70376039	10q21.3	GRCh37/hg19	8825.1	0.918 ± 0.078	1.090 ± 0.174	0.010*
49148631	20q13.13	GRCh37/hg19	8823.3	1.071 ± 0.055	1.275 ± 0.260	0.026*
11979704	19p13.2	GRCh37/hg19	8122.9	0.946 ± 0.072	1.132 ± 0.194	0.011*

Fold change value indicates relative hybridization signal changes between HCC and CCA groups. A copy number ratio = 1 indicates chromosomal material in tumor samples is similar to non-tumor samples. A copy number ratio < 1 indicates chromosomal loss in the tumor samples compared to the non-tumor samples and a copy number ratio > 1 indicates chromosomal gain in the tumor samples compared to the non-tumor samples.

**Table 5:** Distinct chromosomal patterns in HCC and CCA: analysis of top 10 low p-value chromosome regions.

Position	Chromosome region	*P*-value (From bioinformatics tools)	Human genome version	The copy number ratio between HCC and non-tumor samples (Mean±SD)	The copy number ratio between CCA and non-tumor samples (Mean±SD)	*P*-value
89759124	7q21.13	1.4E-9	GRCh37/hg19	1.067 ± 0.037	1.340 ± 0.072	< 0.001*
89765374	7q21.13	3.82E-8	GRCh37/hg19	1.075 ± 0.046	1.290 ± 0.061	< 0.001*
145644305	5q32	3.9E-8	GRCh37/hg19	0.840 ± 0.044	1.036 ± 0.051	< 0.001*
27240723	21q21.3	9.7E-8	GRCh37/hg19	1.028 ± 0.048	1.217 ± 0.051	< 0.001*
40227324	7p14.1	1.19E-7	GRCh37/hg19	1.079 ± 0.059	1.339 ± 0.080	< 0.001*
75263013	7q11.23	1.28E-7	GRCh37/hg19	0.944 ± 0.046	1.176 ± 0.079	< 0.001*
43508513	7p13	1.44E-7	GRCh37/hg19	1.043 ± 0.040	1.201 ± 0.049	< 0.001*
89768961	7q21.13	1.44E-7	GRCh37/hg19	1.050 ± 0.041	1.283 ± 0.083	< 0.001*
145642765	5q32	1.91E-7	GRCh37/hg19	0.825 ± 0.050	0.997 ± 0.041	< 0.001*
5989905	7p22.1	3.09E-7	GRCh37/hg19	0.912 ± 0.076	1.184 ± 0.075	< 0.001*

A copy number ratio = 1 indicates chromosomal material in tumor samples is similar to non-tumor samples. A copy number ratio < 1 indicates chromosomal loss in the tumor samples compared to the non-tumor samples and a copy number ratio > 1 indicates chromosomal gain in the tumor samples compared to the non-tumor samples.

### 3.5 Fluorescence in situ hybridization result

**Fig 6:**
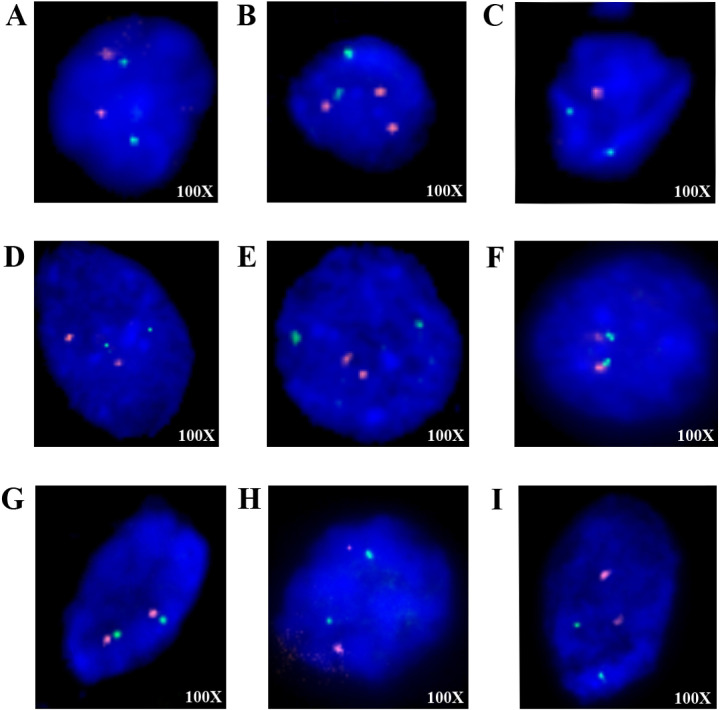
HCC and CCA cases were validated using the FISH technique. (A) HCC case showed a negative result for 1p36 loss or gain with normal nuclei containing two orange and two green signals. (B) HCC case displayed a gain of 1p36 indicated by three orange and two green signals. (C) CCA case exhibited a loss of 1p36 characterized by one orange signal and two green signals. (D-F) Proliferative hepatocytes cases with normal nuclei. (G-I) Cholangitis cases with normal nuclei.

FISH analysis revealed 1p36 chromosomal aberrations in HCC, as illustrated in representative images (Fig 6A and 6B). Fig 6A shows a normal 1p36 signal pattern, indicating no copy number change, whereas Fig 6B demonstrates a gain of the 1p36 region. Conversely, CCA samples exhibited a loss of 1p36 (Fig 6C), aligning with the known CNV profiles. Furthermore, normal 1p36 copy numbers were observed in proliferative hepatocytes (Fig 6D–6F) and within cholangitis nuclei (Fig 6G–6I).

## 4. Discussion

Despite their overlapping histopathological features, HCC and CCA require distinct therapeutic approaches for advanced-stage patients. This study employed CMA to identify chromosomal abnormalities that could aid in the differential diagnosis of these two primary liver cancers. Our findings revealed distinct chromosomal aberration patterns that can be useful for distinguishing between these two types of primary liver cancers.

In this study, HCC patients frequently exhibited gains in chromosomal regions such as 1p36.32, 3q21.3, 4p16.1, and 11p15.5 consistent with other reports [30–32]. Furthermore, we also found gains of 1q23.3-q24.1 and 5q31.1. Like our study, losses were commonly observed at 2p15, 3p11.1-q11.1, 4q12, 5p12-q11.1, 7q11.23, 14q23.2, 17p11.2, 17p13.3, 22q12.1, 22q12.2-q12.3 and 22q13.2. The losses of 4q and 17p have been previously reported using polymerase chain reaction (PCR) and comparative genomic hybridization (CGH) techniques [31,32]. These studies suggest that these regions are associated with molecular cytogenetic changes in HCC. In contrast, chromosome gains in CCA patients were found at 1q32.2, 5p13.2, 8p12-p11.23, 8p22, 8q12.1, 19p13.2, 20p11.21, 20q13.33, and 22q11.22, whereas losses at 1q31.1, 3p25.3, 4q13.2, 9p24.3, 17q12 and 17q21.33. The gains of 5p, 8p, 19p and losses of 1q, 3p and 17q which similar from several studies [19,33–35]. Abnormalities in chromosomes 1, 3 5, 8, 17 and 19 are described in several cancers such as melanoma, colorectal cancer, neuroblastoma, lung cancer, multiple myeloma, renal cell carcinoma, and oral squamous cell carcinoma (OSCC) [36–42]. Our findings revealed that the number of CNVs in HCC is higher than in CCA which may reflect greater genomic instability in this cancer that is consistent with its tumor progression, clinical behavior, and aggressiveness [28,35]. In addition, certain chromosomal regions such as 2q14.2 and 8q24.3 exhibited gains in both HCC and CCA similar to study by Koo et al., suggesting potential shared genomic alterations between these cancers [19]. Interestingly, chromosome 8q24 contains *MYC* gene which plays a role in the tumorigenesis of solid tumors. The co-expression of c-myc and transforming growth factor (TGF) may act as a survival factor for neoplastic cells, accelerating the neoplastic process and significantly promoting the development of cancer [43].

Heatmap analysis further highlighted significant differences in chromosomal marker patterns between HCC and CCA including 2q14.2, 4p16.3, 5q32, 7p14.3, 7p22.1, 7q11.21, 7q11.23, 7q21.3, 7q22.1, 10q21.3, 18q23, 19p13.2, 19q13.2, 21q21.3, 21q22.13, 22q11.21, and 22q12.2. For example, the 4p16.3 region was consistently gained in HCC, while regions at 7p22.1, 19p13.2, 19q13.2, and 22q11.21 were more frequently gained in CCA. These findings suggest that specific chromosomal regions could serve as key indicators for distinguishing between HCC and CCA. Additionally, fold change and p-value analysis showed that regions such as 2q12.3, 7p13, 7p14.1, 7q21.13, 11q13.4, and 20q13.13 had higher signal intensities in CCA than in HCC along with the differences in CNVs levels. Our study identified several candidate chromosomal regions for differential diagnosis, including 1p36.32, 19p13.2, and 19q13.2. Loss of 1p36, a region containing the tumor suppressor gene RUNX3, was predominant in CCA consistent with various studies that revealed the deletion of this region in CCA, OSCC, and adenoid cystic carcinoma [44–46]. RUNX3 plays a crucial role in TGF-β signaling via binding with Smad family including Smad2, Smad3 and Smad4 and tumor suppression pathways. Previous studies have reported that DNA copy number loss and promoter hypermethylation in this region lead to loss of RUNX3 expression in intrahepatic cholangiocarcinoma (iCCA), particularly in cases associated with liver fluke infection [47].

Furthermore, loss of 19p13.2, a region containing genes such as ZNF44, ZNF443, and MAN2B1, was frequently observed in HCC. These genes have been implicated in solid tumors such as neuroblastoma, glioma, and glioblastoma. Additionally, our findings demonstrated that copy number gain at 19q was observed in both HCC and CCA. Previous studies have suggested that genes located in this region, such as ZNF217, GPC3, and CYP2S1, could contribute to the development and progression of cancer [48–50]. Our results indicate that the regions 1p36.32, 19p13.2, and 19q13.2 could serve as biomarkers for distinguishing between HCC and CCA in cases with overlapping histopathological features that would improve early and accurate diagnosis. Furthermore, these chromosome abnormality regions are linked to key pathways involved in the development of HCC and CCA suggesting their regions might serve as potential therapeutic targets for more personalized treatment options.

When we validated the 1p36 region, one of the candidate chromosomal regions for differential diagnosis of HCC and CCA using the fluorescence in situ hybridization (FISH) technique on samples with known CNV profiles. Our results identified a gain of 1p36 in HCC and a loss of 1p36 in CCA which correlated with the CMA results while demonstrating the expected normal nuclear patterns in non-neoplastic cells ensuring assay reliability.

FISH is one of a highly sensitive molecular cytogenetic techniques used to detect both numerical and structural chromosomal abnormality. This method is based on the binding of fluorescently labeled probe to specific chromosome region of interest, allowing visualization in both metaphase and interphase cells under a fluorescence microscope [51]. Unlike conventional karyotyping, FISH offers higher resolution, rapid analysis, and the ability to detect submicroscopic chromosomal alterations making it a valuable tool for confirming chromosomal abnormalities identified through other genomic techniques such as CMA [52]. As expected, these findings confirm the presence of chromosomal alterations in HCC and CCA consistent with Gross et al., who previously reported 1p36 loss in CCA using FISH [53].

In conclusion, the chromosomal abnormality patterns identified in this study may assist in confirming cases with overlapping histopathological features between HCC and CCA contribute to the development of diagnostic tools, and could also be valuable for targeted therapies. The generalizability of this study is limited by its small sample size and reliance on surgically resected specimens. To validate these findings and refine the chromosomal abnormality patterns that differentiate these cancers, further studies are necessary. These studies should involve larger cohorts and employ alternative techniques like fluorescence in situ hybridization (FISH) on cytology samples or cell-free DNA (cfDNA) analysis via digital PCR in liquid biopsies.

## Supporting information

S1 Fig The whole genome view of hepatocellular carcinoma tissues. The log2 ratio data (blue line signal) of each probe marker in the figure indicated chromosomal aberrations. Regions with log2 ratios greater than 0 represented chromosomal gains, while those with log2 ratios less than 0 indicated chromosomal losses.
(TIF)

S2 Fig The whole genome view of cholangiocarcinoma tissues. The log2 ratio data (blue line signal) of each probe marker in the figure indicated chromosomal aberrations. Regions with log2 ratios greater than 0 represented chromosomal gains, while those with log2 ratios less than 0 indicated chromosomal losses.
(TIF)

S1 Table The etiologies involved in the development of HCC and CCA for each patient. (XLSX)

S2 Table The copy number state of each chromosomal region in all HCC and CCA patients. (XLSX)

S3 Table The fold change data for the top 50 chromosome probe markers, including their log2 ratios and transform to copy number variations, in each individual case. (XLSX)

S4 Table The p-values and log2 ratio of the top 50 chromosome probe markers and transform to copy number variations for each patient. (XLSX)
