# Supplementary figures and images for: Distinct chromosome abnormality patterns for differential diagnosis of hepatocellular carcinoma and cholangiocarcinoma
Source: PLoS One. 2025 May 12;20(5):e0322408. doi: 10.1371/journal.pone.0322408 (PMC12068623; doi:10.1371/journal.pone.0322408)

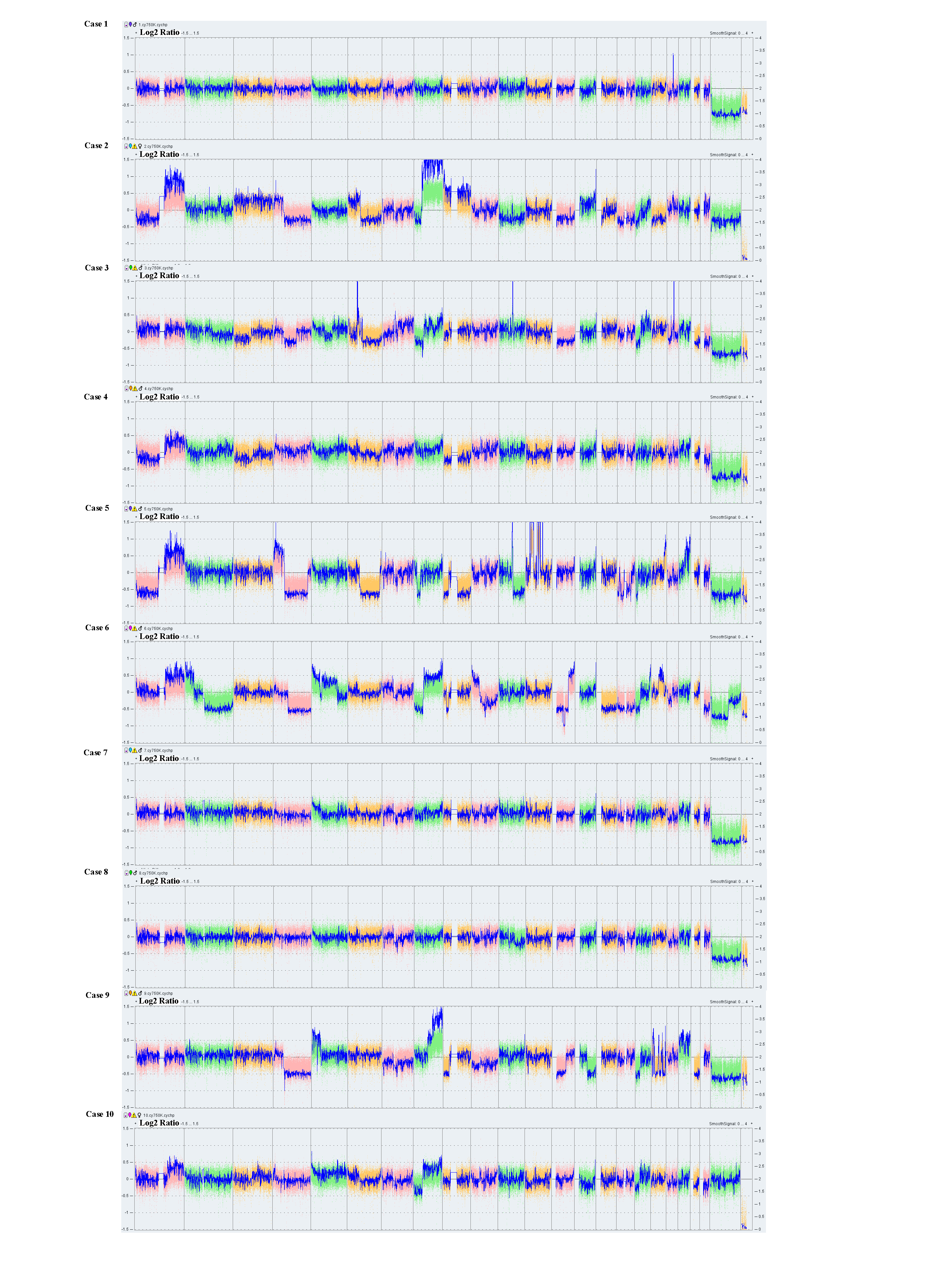

Supplement: S1 Fig — The log2 ratio data (blue line signal) of each probe marker in the figure indicated chromosomal aberrations. Regions with log2 ratios greater than 0 represented chromosomal gains, while those with log2 ratios less than 0 indicated chromosomal losses. (TIF) [file pone.0322408.s001.tif]

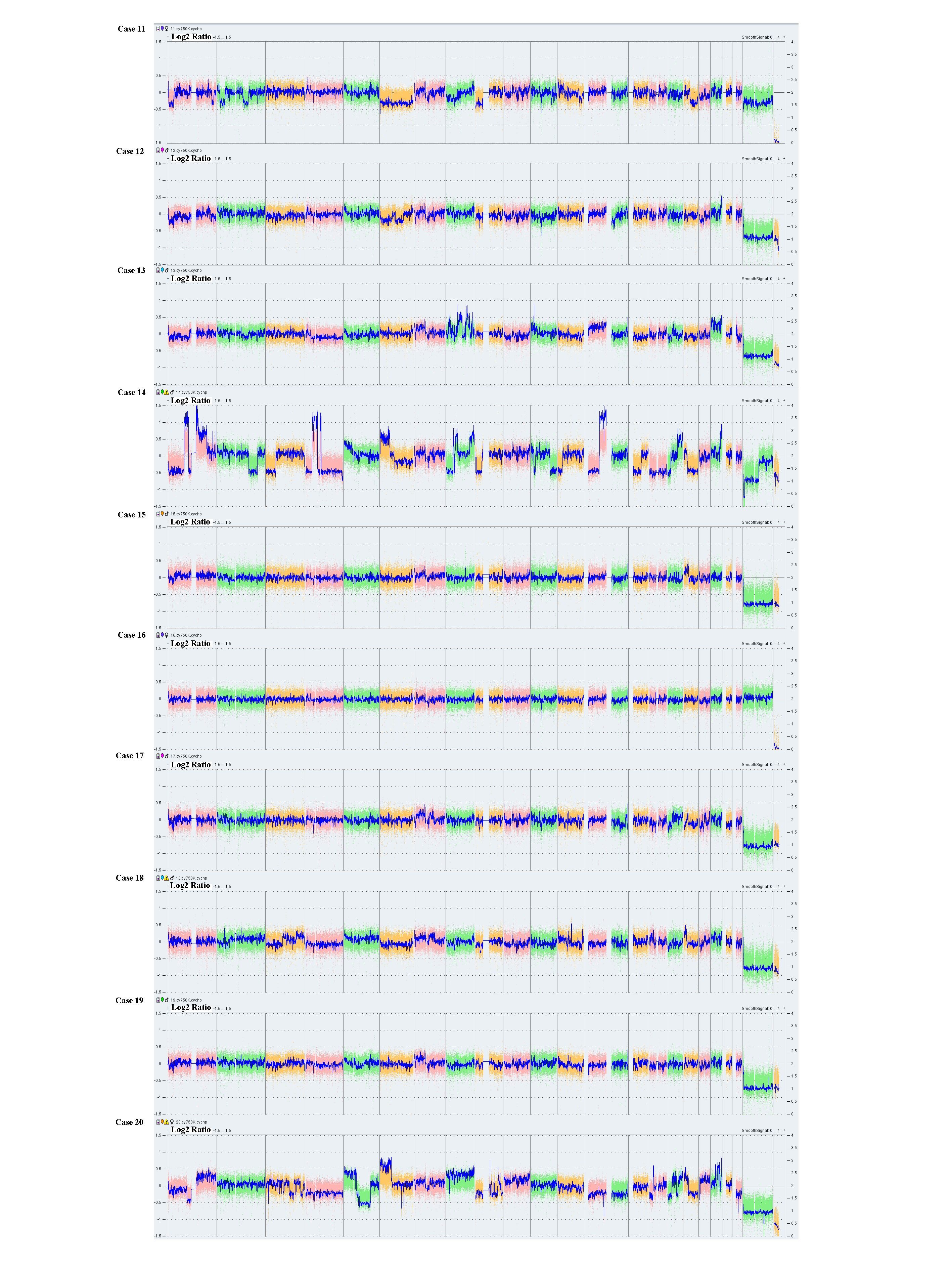

Supplement: S2 Fig — The log2 ratio data (blue line signal) of each probe marker in the figure indicated chromosomal aberrations. Regions with log2 ratios greater than 0 represented chromosomal gains, while those with log2 ratios less than 0 indicated chromosomal losses. (TIF) [file pone.0322408.s002.tif]
